# Discovery of cashmere goat (*Capra hircus*) microRNAs in skin and hair follicles by Solexa sequencing

**DOI:** 10.1186/1471-2164-14-511

**Published:** 2013-07-28

**Authors:** Chao Yuan, Xiaolong Wang, Rongqing Geng, Xiaolin He, Lei Qu, Yulin Chen

**Affiliations:** 1 College of Animal Science and Technology, Northwest A&F University, Yangling, Shaanxi, People’s Republic of China; 2 College of Life Science and Technology, Yancheng Teachers University, Yancheng, People’s Republic of China; 3 College of Life Science, Yulin University, Yulin, People’s Republic of China

**Keywords:** Cashmere goat, MicroRNAs, Hair cycle, Hair follicle, Skin, Solexa sequencing

## Abstract

**Background:**

MicroRNAs (miRNAs) are a large family of endogenous, non-coding RNAs, about 22 nucleotides long, which regulate gene expression through sequence-specific base pairing with target mRNAs. Extensive studies have shown that miRNA expression in the skin changes remarkably during distinct stages of the hair cycle in humans, mice, goats and sheep.

**Results:**

In this study, the skin tissues were harvested from the three stages of hair follicle cycling (anagen, catagen and telogen) in a fibre-producing goat breed. In total, 63,109,004 raw reads were obtained by Solexa sequencing and 61,125,752 clean reads remained for the small RNA digitalisation analysis. This resulted in the identification of 399 conserved miRNAs; among these, 326 miRNAs were expressed in all three follicular cycling stages, whereas 3, 12 and 11 miRNAs were specifically expressed in anagen, catagen, and telogen, respectively. We also identified 172 potential novel miRNAs by Mireap, 36 miRNAs were expressed in all three cycling stages, whereas 23, 29 and 44 miRNAs were specifically expressed in anagen, catagen, and telogen, respectively. The expression level of five arbitrarily selected miRNAs was analyzed by quantitative PCR, and the results indicated that the expression patterns were consistent with the Solexa sequencing results. Gene Ontology and KEGG pathway analyses indicated that five major biological pathways (Metabolic pathways, Pathways in cancer, MAPK signalling pathway, Endocytosis and Focal adhesion) accounted for 23.08% of target genes among 278 biological functions, indicating that these pathways are likely to play significant roles during hair cycling.

**Conclusions:**

During all hair cycle stages of cashmere goats, a large number of conserved and novel miRNAs were identified through a high-throughput sequencing approach. This study enriches the *Capra hircus* miRNA databases and provides a comprehensive miRNA transcriptome profile in the skin of goats during the hair follicle cycle.

## Background

The mammalian hair follicle (HF) is a unique, highly regenerative neuroectodermal-mesodermal interaction system, containing a large number of stem cells [1]. The HF cycles throughout the entire life of mammals to produce new hair through stages of growth (anagen), regression (catagen) and quiescence (telogen) [2]. The HF transition between different stages is driven by a strictly controlled interaction of numerous growth stimulatory and inhibitory factors, which originate from the skin epithelium and mesenchyme [3]. Each stage is characterised by specific patterns of gene activation and silencing [4-6]. These conversions are controlled by the local signal environment, cytokines, hormones, neurotransmitters, as well as the transcription factors and enzymes that are recognised by key mediators in the HF cycle [2,7].

MicroRNAs (miRNAs) are a large family of endogenous, non-coding RNAs, about 22-nucleotide (nt) long, which regulate gene expression through sequence-specific base pairing with target mRNAs [8]. Approximately 25,000 miRNAs have been identified in 193 species of animals, plants and microorganisms. Over the past decade, accumulating evidence has shown that miRNAs play fundamental roles in the development, function, and maintenance of tissues and cells in various organisms [9]. miRNAs are involved in the control of each stage of the hair cycle and regulate the transition between distinct hair-cycle stages by targeting different signalling pathways and transcription factors. Mice carrying a keratinocyte-specific Dicer deletion have severe alterations in HF morphogenesis, formation of large germ-like cysts, and hyperproliferation of the epidermis [10,11]. miR-203 regulates the epidermal keratinocyte differentiation and directed repression of p63 expression [12,13]. Moreover, miR-200b and miR-196a have been implicated in the control of HF development as potential targets for the Wnt signalling pathway [14]. The expression of miR-31 markedly increases during anagen and decreases during catagen and telogen. miR-31 is involved in the establishment of an optimal balance of gene expression in the HF, which is required for its proper growth and hair-fibre formation [15].

Cashmere goats have a double coat consisting of the over hair produced by primary HFs and the under hair (cashmere), produced by secondary HFs [16]. The growth of secondary follicles consists of three stages annually: anagen (April-November), catagen (December-January) and telogen (February-March) [17,18].

The Shanbei White cashmere goat (SWCG), a Chinese domestic goat breed, is farmed to provide cashmere, wool and meat. Here, we present a genetic study of the miRNAs in SWCG HFs, and investigate the differential expression of miRNAs in each distinct stage of SWCG HF cycles by Solexa sequencing. We further explore their functions in the regulation of the hair growth cycle.

## Results

### Solexa-sequencing of small RNAs

In order to identify miRNAs involved in the three phrases (anagen, catagen and telogen) of the hair cycle, three small RNA (sRNA) libraries representing the above three phrases were constructed from a mixed pool of ten adult cashmere goat skin samples. The sRNA libraries were subsequently sequenced by Solexa sequencing. A total of 63,109,004 raw reads were obtained. After discarding the sequences shorter than 18 nt, eliminating low-quality sequences and removing contaminants formed by adapter–adapter ligation, reads without 3′ ligation and insert tags were obtained. Collectively, 61,125,752 clean reads remained for further analysis (Table 1).

**Table 1:** The distribution of total small RNA tags by Solexa sequencing

**Type**	**Anagen**		**Catagen**		**Telogen**		**Total**
	**Counts**	**Percent (%)**	**Counts**	**Percent (%)**	**Counts**	**Percent (%)**	
total_reads	16,861,573		18,788,688		27,458,743		63,109,004
high_quality	16,756,965	100	18,729,213	100	27,307,694	100	62,793,872
3′adapter_null	57,530	0.34	5433	0.03	44,385	0.16	107,348
insert_null	103,836	0.62	81,245	0.43	76,673	0.28	261,754
5′adapter_contaminants	30,860	0.18	31,809	0.17	15,778	0.06	78,447
smaller_than_18nt	566,742	3.38	498,627	2.66	154,658	0.57	1,220,027
polyA	169	0.00	187	0.00	188	0.00	544
clean_reads	15,997,828	95.47	18,111,912	96.70	27,016,012	98.93	61,125,752

**Figure 1:**
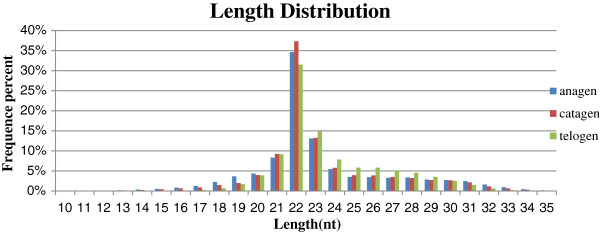
Sequence length distribution of the clean reads based on three total abundance and distinct sequences.

We then analysed the length distribution based on the three libraries and distinct sequences to assess the sequencing quality (Figure 1). Among these sequences, most were distributed in the 18–30 nt range. The highest percentages of these sRNAs were 22-nt long, which is consistent with the common size of miRNAs.

Subsequently, in order to analyse their expression and distribution in the goat genome, all of the clean Solexa reads were aligned with the goat genome sequence using SOAP software (Additional file 1: Figure S1). Of 15,997,828 reads screened in the anagen stage, 10,791,973 reads and 598,873 unique sRNAs, representing 67.46% of total reads and 45.76% of unique sRNAs, respectively, were matched by the goat genome sequence (Additional file 1: Figure S1A). To further assess the efficiency of Solexa sequencing for miRNA detection, all of the clean reads were annotated and classified using tag2annotation software (developed by Beijing Genomics Institute (BGI)), aligned against the Rfam10.1 database and the miRBase19.0 database. However, some sRNA reads may be mapped to more than one category. In order to better align every unique sRNA to one annotation, we conducted the following priority criteria: rRNA etc. (in which Genbank > Rfam) > conserved miRNA > repeat > exon > intron.

The total rRNA proportion is a sign of the quality of the samples, for instance, the proportion of total rRNA should be less than 60% in plant samples [19], and 40% in animal samples (unpublished data by BGI). The total rRNA proportion in the present study was 36.5, 30.47 and 28.01% in anagen, catagen and telogen, respectively, indicating that the skin samples used were of a high quality. All of the clean reads were divided into the following categories: exon_antisense, exon_sense, intron_antisense, intron_sense, miRNA, rRNA, repeat, scRNA, snRNA, snoRNA, srpRNA, tRNA, unan (unannotated) (Additional file 1: Figure S1A). Among them, the conserved miRNAs have 1,737,508 total reads and 2841 unique reads, which represented 10.86% of total reads and 0.22% of unique clean reads. Of the unique reads, 56.89% were identified as potential novel miRNAs, representing 24.11% of clean reads.

### Expression analysis of conserved miRNAs

**Figure 2:**
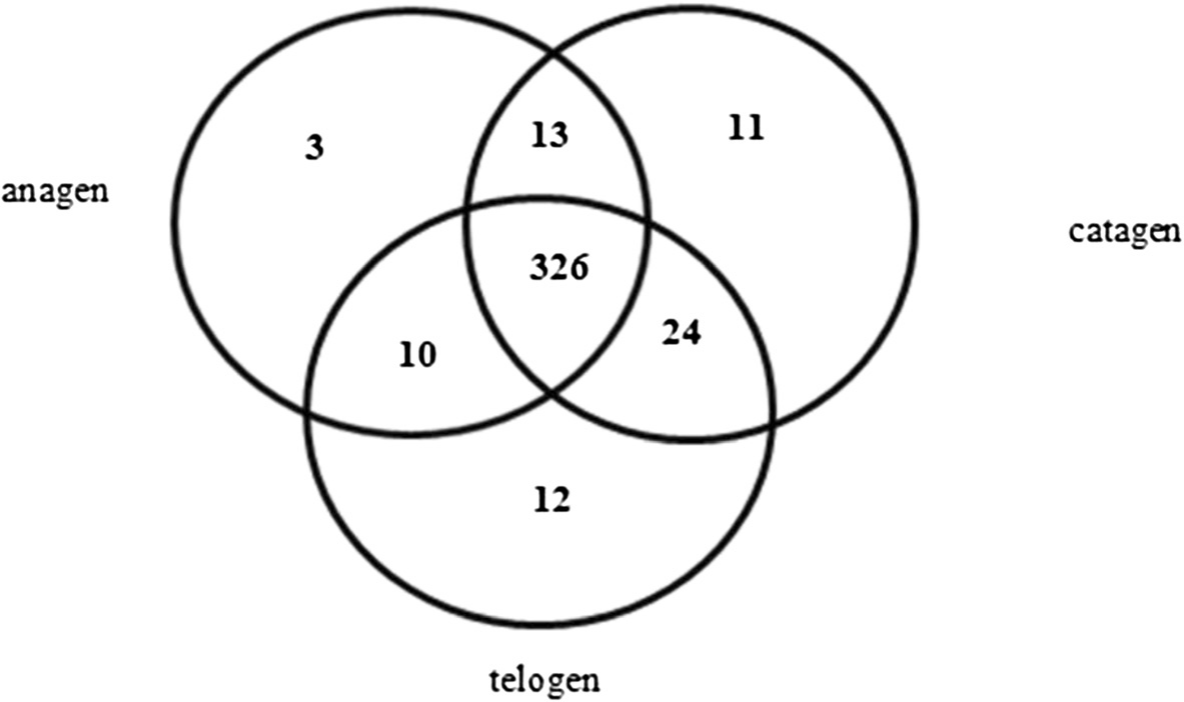
**Venn diagram of differentially expressed conserved miRNAs at the three stages of HF cycling in cashmere goats.** Numbers in parentheses are numbers of differentially expressed miRNAs at each stage.

Since there are no goat miRNAs available in the miRbase 19.0 database, we compared the clean reads with the miRNA precursor/mature miRNAs with known cattle sequences. Our results demonstrate that miRNA expression is abundant in the skin of cashmere goats, as a total of 399 miRNAs were found in the three stages of the HF cycle (Additional file 2: Figure S2). Among them, 326 miRNAs were expressed in all three cycling stages, whereas 3, 12 and 11 miRNAs were specifically expressed in anagen, catagen, and telogen, respectively (Figure 2).

**Figure 3:**
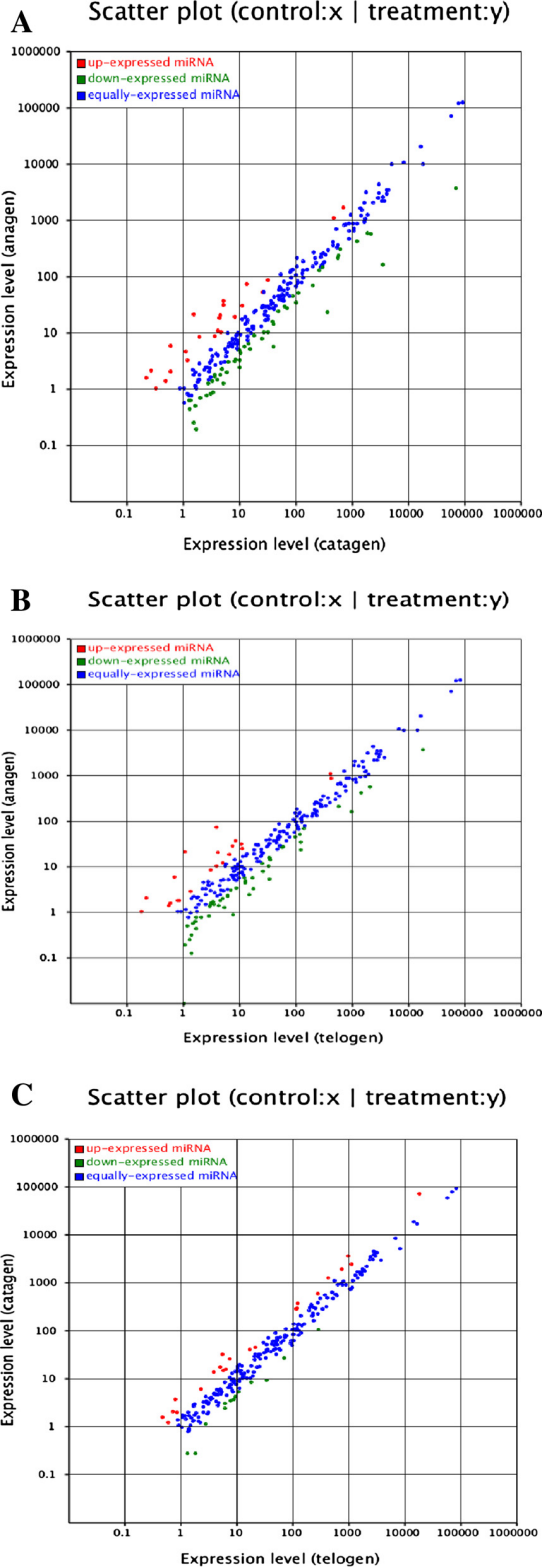
**Differences of miRNA expression between the samples from every two hair cycle stage.** Each point represents an miRNA. The X and Y axes show the expression level of miRNAs in every two samples, respectively. Red points represent miRNAs with a ratio > 2, Blue points represent miRNAs with 1/2 < ratio < =2, Green points represent miRNAs with ratio < = 1/2, Ratio = Normalised expression in Treatment/Normalised expression in Control. **(A**) catagen-anagen; **(B)** telogen-anagen; **(C)** telogen-catagen.

**Figure 4:**
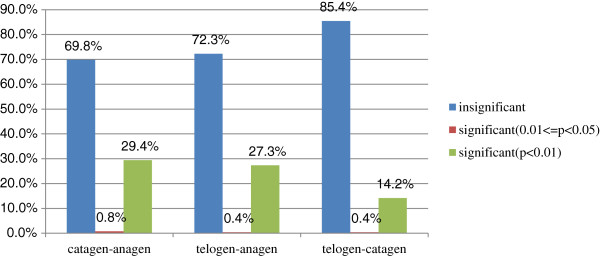
Changes in miRNA expression among different hair cycle stages.

We then analysed the differentially expressed miRNAs between the samples from every two-hair-cycle stage (Figure 3, Additional file 3: Figure S3). Most of the expression levels were equivalent, but there were also some miRNA expression differences between the two stages (Figure 3). 68.9% of the miRNA expression was not significant, 0.8% of the miRNAs were significantly different (0.01 ≤ p < 0.05) and 29.4% of the miRNAs were significantly different (p < 0.01) in the catagen and anagen stages (Figure 4).

**Figure 5:**
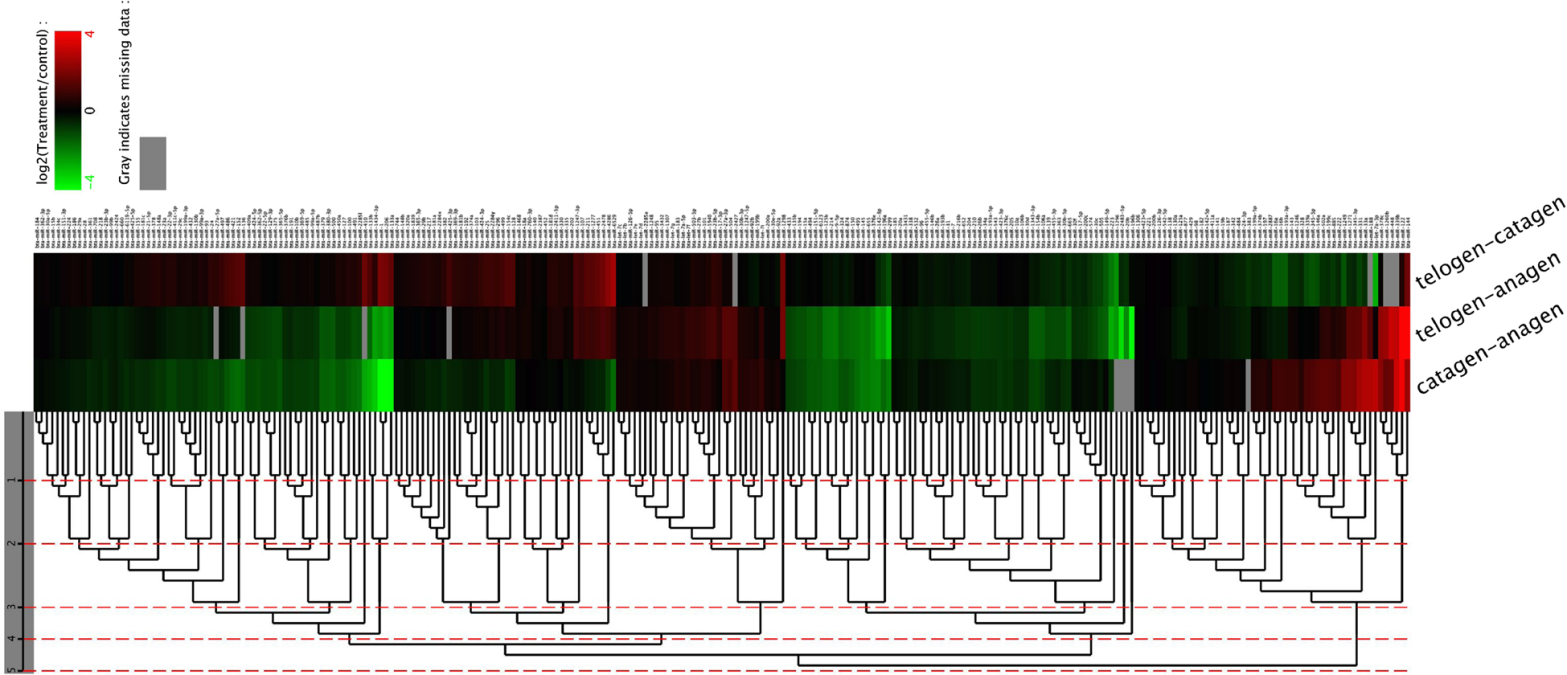
**Clustering of miRNAs differentially expressed during HF Cycling.** Red indicates that the miRNA has a higher expression level in the treatment samples; green indicates that the miRNA has a higher expression in the control samples and gray indicates that the miRNA has no expression in at least one sample. Each row in the figure represents one miRNA, and each column shows one sample pair. Each cell shows the differential expression of a miRNA in one sample pair. Heat map represents differentially expressed miRNAs between distinct stages of the hair cycle. Colour map is used to visualise the difference in expression.

miRNAs with similar expression patterns in different sample pairs were clustered together. Clustering analysis was based on the sample difference model by using Cluster software, and the results were viewed with Java Treeview. All differentially expressed miRNAs clustered together after five rounds of clustering (Figure 5).

### Quantitative RT-PCR validation

**Figure 6:**
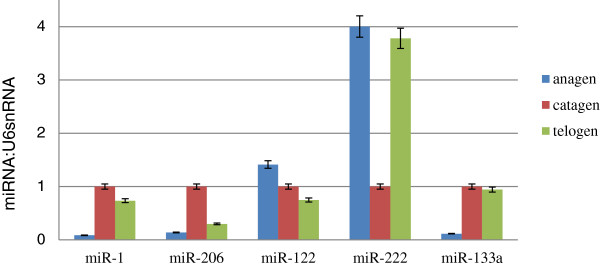
**qPCR validation of miRNA expression in skin samples among different hair cycle stages.** The abundance of miR-1, miR-206, miR-122, miR-222, and miR-133 were normalised relative to the abundance of U6 small nuclear RNA (snRNA). Error bars show the 95.0% confidence interval of the mean.

To verify the Solexa sequencing data, we randomly selected five differentially expressed miRNAs (miR-1, miR-206, miR-122, miR-222, and miR-133), and conducted quantitative RT-PCR. The relative expression levels of five selected miRNAs were consistent with the Solexa sequencing results since they had a similar trend of expression in all three periods (Figure 6).

### Identification of novel miRNAs

The characteristic hairpin structure of miRNA precursors can be used to predict novel miRNAs. We predicted novel miRNAs by exploring the secondary structure, the Dicer cleavage site and the minimum free energy of the unannotated small RNA reads, which could be mapped to goat genome sequences by using Mireap software. In total, 15,592,654 unannotated sequences were used to predict novel miRNAs by using the Mireap software. Among 172 potential novel miRNAs identified, 36 miRNAs were expressed in all three cycling stages, whereas 23, 29 and 44 miRNAs were specifically expressed in anagen, catagen, and telogen, respectively (Additional file 4: Figure S4, S4-1and S4-2). In addition, the length of the novel miRNA sequences ranged from 20 to 24 nt, with a distribution peak at 22 nt and their 5' ends were comprised most frequently of uridine (U) (Additional file 4: Figure S4, S4-3).

### Target gene prediction for miRNAs

miRNAs negatively regulate gene expression by base pairing between the 5' end of the miRNA (i.e., 2–8 nt, the “seed” region) and the 3′ untranslated regions (3'UTR) of target mRNAs [8,15,20-22]. Mireap software was used to predict target genes of the miRNA by searching the bovine reference gene database (http://hgdownload.cse.ucsc.edu/goldenPath/bosTau7/bigZips/refMrna.fa.gz). In the anagen stage, 750,038 target sites in 13,860 target genes were predicted for 352 miRNAs, whereas 796,849 target sites in 13,867 target genes were predicted for 372 miRNAs in catagen, and 803,957 target sites into 13,864 target genes were predicted among 374 miRNAs in telogen.

### Gene Ontology (GO) enrichment and KEGG pathway analysis of target genes

GO enrichment analysis is used for predicting candidate target genes of miRNAs. GO enrichment analysis for target genes based on the cellular component showed that 10,830 genes were termed good or better than 1 using the Component Ontology with *p*-value analysis (Additional file 5: Figure S5). More than 83.9% of genes were clustered into the cell part, followed by the intracellular part accounting for 76.2% of target genes. Analysis of molecular function showed that 10,178 genes were assigned different functions, specifically 81.6% of genes were related to binding functions, and 9979 genes were related to biological processes. Most of the genes were involved in cellular or metabolic processes. For example, 79.4% of the genes were involved in cellular processes, and 55.8% and 47.2% of the genes were involved in metabolic processes and biological regulation, respectively.

KEGG pathway annotation showed that 10,563 target genes were annotated for 278 biological functions. Most of these genes were involved in cellular metabolism, diseases and signal transduction (Additional file 6: Figure S6). The most commonly indicated pathway was the Metabolic pathways, with 1213 genes representing 11.49% of the total target genes, followed by the Pathways in cancer (3.26%), MAPK signalling pathway (3%), Endocytosis (2.68%), and Focal adhesion (2.66%).

## Discussion

The sRNA digitalisation analysis based on high-throughput sequencing uses the sequencing-by-synthesis (SBS) technology predicts novel miRNAs and constructs the sRNA differential expression profile between samples from every two-hair-cycle stage, which could be used as a powerful tool for the functional studies of sRNA [23-26].

In this study, objective preliminary analysis of three cDNA libraries has shown that 22-nt sRNA is the major type of sRNA, which is consistent with the majority of sRNA-lengths in cattle [23], fish [24], goats [25,26], swine [27] and chickens [28]. Mature miRNAs, which are identical to the classical size of Dicer cleavage products [29], also have a similar trend. However, the major type of sRNA screened by Solexa sequencing in wheat is 24-nt in length [30], implying that there are length differences between miRNAs from animal and plant species.

Several HF miRNAs have been shown to be involved in the regulation and forming of hair loss, hypertrichosis and skin diseases in mice and humans [1,8], however, rarely have been performed in goats for fibre. Of the nine miRNAs were specifically expressed in cashmere goat dorsal skin [18], only four of them were examined in the present study: miR-1, miR-374, miR-455-3p and miR-92b. This discrepancy might be caused by inaccurate processing, base modification and sequencing/PCR errors, and analysis used inconsistencies of the database. Moreover, different genome assembly (bovine vs. caprine) may lead to the identification of various miRNAs, even in the same goat breed. For instance, compared with 352 conserved miRNAs and 83 novel miRNAs identified in the present study, Liu et al. (2012) discovered 316 conserved miRNAs and 22 novel miRNAs in another Chinese cashmere goat breed (Aerbasi White Cashmere Goat) by deep sequencing skin tissues that represented the anagen stage [26].

In the whole hair cycle, the abundance of expression of let-7a-5p, let-7f, let-7b, let-7c, let-7g, miR-199a-3p, miR-143, miR-1, and miR-320a reached their highest levels in the present study (Table 2). These miRNAs are involved in cell differentiation [31,32], and proliferation [33], and the development of nerves [34], heart [35], lung [36] and muscle [37,38], suggesting that these miRNAs may play major roles in the regulation of fundamental biological processes, as well as the development of skin and HF.

**Table 2:** Highly expressed miRNAs (top 10) in the three stages of HF cycling

**Anagen**		**Catagen**		**Telogen**	
**miRNA**	**count**	**miRNA**	**count**	**miRNA**	**count**
bta-let-7a-5p	1,975,152	bta-let-7a-5p	1,661,018	bta-let-7a-5p	2,270,869
bta-let-7f	1,886,542	bta-let-7f	1,423,572	bta-let-7f	1,885,418
bta-let-7b	1,131,783	bta-miR-1	1,273,960	bta-let-7b	1,563,491
bta-let-7c	324,995	bta-let-7b	1,069,133	bta-miR-1	496,437
bta-let-7 g	168,427	bta-miR-199a-3p	335,203	bta-let-7c	452,270
bta-miR-199a-3p	159,586	bta-let-7c	306,476	bta-miR-199a-3p	391,284
bta-miR-143	155,737	bta-let-7 g	149,045	bta-miR-143	224,149
bta-miR-101	68,706	bta-miR-143	90,997	bta-let-7 g	185,293
bta-miR-1	58,142	bta-miR-103	80,670	bta-miR-26a	103,535
bta-miR-320a	55,595	bta-miR-320a	74,766	bta-miR-320a	87,364

HFs undergo a process of cyclical regeneration: growth, regression and quiescence. The transition from one stage to another is regulated by abundant molecules. Our results showed that the expression patterns of miR-1, miR-133a, miR-133b, miR-144, miR-206, miR-299, miR-331 and miR-4286 (Additional file 3: Figure S3) were significantly different in the three stages (p < 0.01), indicating that they may participate in the regulation of follicular transition.

 According to the results of our Cluster analysis, the expression patterns of miRNAs in cashmere goat skins could be divided into five types during hair cycling (Figure 5). One of the patterns is the miRNA expression level increases at anagen and then decreases during catagen and telogen (such as miR-502a, -199c, -885, -222, -1249, -1271, -345-3p). In comparison with telogen and catagen, most of the miRNAs demonstrated dramatic expression changes in the anagen stage of HFs and skin, implying that these miRNAs probably participate in the formation of new hair shafts and activation of a large number of signalling pathways controlling the expression of genes encoding hair-specific molecules [2,15].

The major pathways predicted in this study were also mentioned previously. The findings of HF signal pathways in humans and mice indicates that the Wnt [39], TGF-β [40], MAPK [41], Shh [42], Notch and JAK-STAT [43] pathways widely participate in every part of the HF cycle, development, and morphogenesis, and greatly contribute to all kinds of HF. Of the target genes identified, 3% were from the MAPK pathway, 1.52% from Wnt, 0.87% from TGF-β, 0.42% from Shh, 0.43% from Notch and 1.37% from JAK-STAT. The miRNAs that correspond to these target genes will be our main candidate miRNAs for further studies on hair cycles.

## Conclusions

During the anagen-catagen-telogen transformation of the hair cycle in cashmere goats, 399 conserved miRNAs and 172 novel miRNAs were found via a high-throughput sequencing approach. Our findings enrich the caprine miRNA databases and provide new insights into the miRNA transcriptome in cashmere goat skin and the HF cycle.

## Methods

### Animal and sample preparation

Approximately 1-cm^2^ skin samples were harvested from the side of the body of adult goats at distinct hair cycle stages (anagen, catagen and telogen) in SWCG (five males and five females), frozen in liquid nitrogen and stored at −80°C for analysis. All the experimental procedures with goats used in the present study had been given prior approval by the Experimental Animal Manage Committee of Northwest A&F University under contract (2011–31101684).

### Small RNA library construction and sequencing

Total RNA from the mixed skin tissues of ten adult goats was isolated using the RNAiso plus kit (TaKaRa, Dalian, China) according to the manufacturer’s protocol. The RNA quality and quantity were determined using an Agilent 2100 Bioanalyzer (Agilent, CA, USA). Small RNA fragments of 18–30 nt in length were isolated and purified from total RNA using 15% denaturing polyacrylamide gel electrophoresis (PAGE). Subsequently, a 3′ RNA adaptor and 5' RNA adaptor were ligated to the RNA pool using T4 RNA ligase. The sRNAs ligated with adaptors were subjected to RT-PCR amplification, and the cDNA was further amplified. The PCR products were purified using 10% PAGE to construct an sRNA library. The sRNA libraries were constructed from skin tissue from the anagen, catagen and telogen stages, and were sequenced using an Illumina/Solexa 1G Genome Analyzer at the BGI, Shenzhen.

### Sequence analysis

The basic figures from sequencing were converted into sequence data by base calling. After removing low quality reads and reads with 5′ primer contaminants, reads without 3′ primer, reads without the insert tag, reads with poly (A), and reads shorter than 18 nt, the clean reads were obtained. We then summarised the length distribution of these clean reads. The clean reads that were obtained were compared with the ncRNAs (rRNAs, tRNAs, snRNAs, and snoRNA) deposited in the NCBI GenBank database and the Rfam10.1 database using BLAST to annotate the sRNA sequences. The clean reads were mapped to the goat genome (http://goat.kiz.ac.cn/GGD/download.htm) by SOAP v1.11 to analyse their expression and distribution in the genome. The clean reads were aligned against the miRNA precursor/mature miRNA of *Bos taurus* in miRBase19.0 (http://www.mirbase.org/) to identify the conserved miRNAs. The unannotated sequences were used to predict potential novel miRNA candidates by Mireap (http://sourceforge.net/projects/mireap/). For an sRNA to be considered a potential novel miRNA candidate, the predicted sequences should also meet the following parameters according to Mireap: minimal miRNA sequence length (18 nt), maximal miRNA sequence length (26 nt), minimal miRNA reference sequence length (20 nt), maximal miRNA reference sequence length (24 nt), minimal depth of Drosha/Dicer cutting site (3 nt), maximal copy number of miRNAs on reference (20 nt), maximal free energy allowed for a miRNA precursor (−18 kcal/mol), maximal space between miRNA and miRNA* (35 nt), minimal base pairs of miRNA and miRNA* (14 nt), maximal bulge of miRNA and miRNA* (4 nt), maximal asymmetry of miRNA/miRNA* duplex (5 nt), and the flank sequence length of miRNA precursor (10 nt).

The selected sequences were then folded into a secondary structure using the RNA folding program, Mfold 3.2 software. If a perfect stem-loop structure was formed, the sRNA sequence was located at one arm of the stem, and the above criteria were met, the sRNA was considered to be a potential novel miRNA candidate. We predicted the target genes of the miRNA using the Mireap software program based on the following criteria: no more than four mismatches between the sRNA and target (G-U bases count as 0.5 mismatches), no more than two adjacent mismatches in the miRNA/target duplex, no adjacent mismatches in positions 2–12 of the miRNA/target duplex (5′ of miRNA), no mismatches in positions 10–11 of the miRNA/target duplex, no more than 2.5 mismatches in positions 1–12 of the miRNA/target duplex (5′ of miRNA), and the minimum free energy (MFE) of the miRNA/target duplex should be ≥ 75% of the MFE of the miRNA bound to its perfect complement.

### GO enrichment and KEGG pathway analyses

We revealed the functions significantly associated with the predicted target gene candidates of the miRNAs using GO analysis. This method first maps all target gene candidates to GO terms in the database (http://www.geneontology.org/), calculating gene numbers for each term, then uses hyper geometric testing to find significantly enriched GO terms in target gene candidates compared with the reference gene background. The calculating formula is:

$$P=1-\overset{m-1}{\underset{i=0}{\sum }}\frac{\left(\begin{array}{c} M\\ i \end{array}\right)\left(\begin{array}{c} N-M\\ n-i \end{array}\right)}{\left(\begin{array}{c} N\\ n \end{array}\right)}$$

In the formula above, N is the number of all genes with GO annotation; n is the number of target gene candidates in N, M is the number of all genes that are annotated to a certain GO term, and m is the number of target gene candidates in M. We used the Bonferroni Correction for the *p*-value to obtain a corrected *p*-value. GO terms with corrected *p*-values of ≤ 0.05 are defined as significantly enriched in target candidate genes. This analysis is able to recognise the main biological functions for target gene candidates.

Subsequently, the main pathways in which the target candidate genes are involved were revealed by KEGG pathway analysis. The calculating formula is the same as that for GO analysis. Here, N is the number of all genes with a KEGG annotation, n is the number of target gene candidates in N, M is the number of all genes annotated to a certain pathway, and m is the number of target gene candidates in M. Genes with FDR ≤ 0.05 are considered to be significantly enriched in target gene candidates. The KEGG analysis reveals the main pathways involving the target gene candidates.

### Differential expression analysis

Scatter plots were used to demonstrate differentially expressed miRNA between every two follicular stages. The procedures are as follows: (1) The expression of the miRNA in two samples (control and treatment) is normalised to get the expression of transcript per million (TPM). Normalisation formula: Normalised expression = actual miRNA count/total count of clean reads*1000000; (2) The fold-change and *p*-value are calculated from the normalised expression. Then log2ratio plot and scatter plot are generated. Fold-change formula:

$$\text{Fold}\_\text{change}=\text{log}2\;\left(\text{treatment}/\text{control}\right)$$

*p*-value formula:

$$p\left((,x|y)\right)={\left(\frac{N_{2}}{N_{1}}\right)}^{y}\frac{\left(x+y\right)!}{x!y!{\left(1+\frac{N_{2}}{N_{1}}\right)}^{\left(x+y+1\right)}}\;\begin{array}{c} C(\left(y\leq y_{\min}|x)\right)=\overset{y\leq y_{\min}}{\underset{y=0}{\sum }}p\left((,y|x)\right)\\ D(\left(y\geq y_{\max}|x\right))=\overset{\infty }{\underset{y\geq y_{\max}}{\sum }}p\left(y|x\right) \end{array}$$

After normalisation, if the miRNA gene expression amount of both samples is zero, then revise to 0.01, and if the miRNA gene expression amount for both samples is less than 1, these samples do not participate in the differential expression analysis because their expression levels are too low.

### Quantitative RT-PCR

Total RNA from the mixed skin tissues of ten adult goats was isolated using the RNAiso plus kit (TaKaRa, Dalian, China), and real-time quantification of miRNAs was performed by stem-loop RT-PCR. 1 μg of total RNA was reverse transcribed to cDNA using the RevertAid First Strand cDNA Synthesis Kit (Thermo Scientific Fermentas) and stem-loop RT primers (Additional file 7: Figure S7) [44]. The mix was then incubated at 42°C for 60 min and 70°C for 5 min. Real-time PCR was performed using iQ5 (Bio-Rad, Hercules, CA, USA) and a standardised protocol. In a 25 μl reaction mixture, 2.0 μl of cDNA (at a 1:4 dilution) was used for amplification, with 12.5 μl of SYBR Premix Ex Taq^TM^ II (TaKaRa, Dalian, China), 1.0 μl of specific forward primer, 1.0 μl of universal primer, and 8.5 μl of water. The reactions were incubated at 95°C for 3 min, followed by 45 cycles of 94°C for 15 s, 60°C for 30 s and 72°C for 45 s. The abundance of selected miRNAs was normalised relative to that of U6 snRNA. All reactions were performed in triplicate. The threshold cycle (CT) was determined using the default threshold settings and the data was analysed using the 2^–ΔΔCt^ program.

## Abbreviations

miRNA: microRNA; HF: hair follicle; SWCG: Shanbei White cashmere goat; sRNA: small RNA; UTR: Untranslated regions; CDS: Coding sequence; GO: Gene ontology; KEEG: Kyoto encyclopedia of genes and genomes; RT-PCR: Reverse transcription PCR; BGI: Beijing Genomics Institute; MFE: Minimum free energy; TPM: Transcript per million.

## Competing interests

The authors declare that they have no competing interests.

## Authors’ contributions

Conceived and designed the experiments: CY RG YC. Performed the experiments: CY RG. Analyzed the data: CY RG XH. Contributed reagents and materials: YC LQ. Wrote the paper: CY XW. All authors read and approved the final manuscript.

## Supplementary Material

Additional file 1: Figure S1 The flowing results of data filtration and the distribution of sequenced small RNAs. Click here for file

Additional file 2: Figure S2 Conserved miRNAs in the three stages of HF cycling. Click here for file

Additional file 3: Figure S3 Differentially expressed of conserved miRNAs during HF cycling. Click here for file

Additional file 4: Figure S4 Predicted information on the novel cashmere goat miRNAs. Click here for file

Additional file 5: Figure S5 GO enrichment analysis for the target genes of conserved miRNAs. Click here for file

Additional file 6: Figure S6 KEGG pathways for the target genes of conserved miRNAs. Click here for file

Additional file 7: Figure S7 Primers for real time qPCR. Click here for file

## Accession numbers

The sequence data from this study have been deposited at Gene Expression Omnibus (GEO) and can be accessed with the number GSE47742.
